# Preoperative homocysteine modifies the association between postoperative C-reactive protein and postoperative delirium

**DOI:** 10.3389/fnagi.2022.963421

**Published:** 2022-09-21

**Authors:** Xin Ma, Xinchun Mei, Tianyi Tang, Meijuan Wang, Xiaoyi Wei, Hailin Zheng, Jing Cao, Hui Zheng, Kathryn Cody, Lize Xiong, Edward R. Marcantonio, Zhongcong Xie, Yuan Shen

**Affiliations:** ^1^Department of Psychiatry, Shanghai Tenth People’s Hospital, Tongji University School of Medicine, Shanghai, China; ^2^Anesthesia and Brain Research Institute, Tongji University School of Medicine, Shanghai, China; ^3^Massachusetts General Hospital Biostatistics Center, Massachusetts General Hospital and Harvard Medical School, Boston, MA, United States; ^4^Anesthesia Research Center, Department of Anesthesia, Critical Care and Pain Medicine, Massachusetts General Hospital and Harvard Medical School, Boston, MA, United States; ^5^Department of Anesthesiology and Translational Research Institute of Brain and Brain-Like Intelligence, Shanghai Fourth People’s Hospital of Tongji University School of Medicine, Shanghai, China; ^6^Divisions of General Medicine and Gerontology, Department of Medicine, Beth Israel Deaconess Medical Center and Harvard Medical School, Boston, MA, United States; ^7^Geriatric Anesthesia Research Unit, Department of Anesthesia, Critical Care and Pain Medicine, Massachusetts General Hospital and Harvard Medical School, Charlestown, MA, United States; ^8^Shanghai Mental Health Center of Shanghai Jiao Tong University, Shanghai, China

**Keywords:** anesthesia, surgery, postoperative delirium, C-reactive protein, homocysteine

## Abstract

**Background:**

Homocysteine and C-reactive protein (CRP) may serve as biomarkers of postoperative delirium. We set out to compare the role of blood concentration of homocysteine versus CRP in predicting postoperative delirium in patients.

**Materials and methods:**

In this prospective observational cohort study, the plasma concentration of preoperative homocysteine and postoperative CRP was measured. Delirium incidence and severity within 3 days postoperatively were determined using the Confusion Assessment Method and Confusion Assessment Method-Severity algorithm.

**Results:**

Of 143 participants [69% female, median (interquartile range, 25th–75th) age of 71 (67–76) years] who had knee or hip surgery under general anesthesia, 44 (31%) participants developed postoperative delirium. Postoperative plasma concentration of CRP was associated with postoperative delirium incidence [adjusted odds ratio (OR) per one standard deviation change in CRP: 1.51; 95% Confidence Interval (CI): 1.05, 2.16; *P* = 0.026], and severity [in which each one standard deviation increase in postoperative CRP was associated with a 0.47 point (95% CI: 0.18–0.76) increase in the severity of delirium, *P* = 0.002] after adjusting age, sex, preoperative Mini-Mental State Examination score and the days when postoperative CRP was measured. A statistically significant interaction (adjusted *P* = 0.044) was also observed, in which the association between postoperative plasma concentration of CRP and postoperative delirium incidence was stronger in the participants with lower preoperative plasma concentrations of homocysteine compared to those with higher preoperative levels.

**Conclusion:**

Pending validation studies, these data suggest that preoperative plasma concentration of homocysteine modifies the established association between postoperative plasma concentration of CRP and postoperative delirium incidence.

## Introduction

Postoperative delirium, an acute confusion status after anesthesia and surgery, is associated with adverse effects with annual care costs of $ 32.9 billion (Gou et al., 2021). Population studies have demonstrated that patients with delirium may face a 12.5-fold increased incidence of newly diagnosed Alzheimer’s disease (AD) (Fong et al., 2009, 2015; Witlox et al., 2010; Ags/Nia Delirium Conference Writing Group and Faculty, 2015).

Inflammation is emerging as a candidate process propagating postoperative delirium, mainly *via* increased production of pro-inflammatory cytokines in the blood and brain (Wilson et al., 2002; Kalman et al., 2006; Ramlawi et al., 2006a,b; Rudolph et al., 2008). Almost every patient has postoperative inflammation, but not every patient develops postoperative delirium. Thus, inflammation alone may be insufficient to explain postoperative delirium. Instead, patients who develop postoperative delirium may have other changes (predisposing factors) that exacerbate the postoperative inflammation (precipitating factor), leading to postoperative delirium. Therefore, it is crucial to study the contributions of both predisposing and precipitating factors—and their interaction—to the development of postoperative delirium.

Hyperhomocysteine (elevated plasma homocysteine concentration) is a potential predisposing factor owing to its involvement in cardiovascular disease (Refsum et al., 1998) and AD (Morris, 2003). Preoperative plasma hyperhomocysteine concentration is associated with postoperative delirium (Kim et al., 2015; Weerink et al., 2018), but conflicting results exist (Vahdat Shariatpanahi et al., 2019; Guo et al., 2020). Moreover, hyperhomocysteine increases blood-brain barrier (BBB) permeability (Kamath et al., 2006; Beard et al., 2011) and promotes neuroinflammation, which can lead to cognitive dysfunction (Adamis et al., 2009; Varatharaj and Galea, 2017). C-reactive protein (CRP) is associated with postoperative delirium (Dillon et al., 2017; Vasunilashorn et al., 2017). However, it remains largely unknown whether the established association between the postoperative blood concentration of CRP (a precipitating factor) and postoperative delirium depends on preoperative predisposing factors such as preoperative blood homocysteine concentration.

A recent study demonstrated the interaction between gene Apolipoprotein E (a predisposing factor) and postoperative CRP (a protein and precipitating factor) on postoperative delirium (Vasunilashorn et al., 2020). However, the protein-protein interaction of predisposing factors and precipitating factors on the incidence and severity of postoperative delirium has not been investigated.

Therefore, we set out to determine the effects of preoperative plasma concentration of homocysteine, the postoperative plasma concentration of CRP, and their interactions on the incidence and severity of postoperative delirium in patients. It was hypothesized that preoperative plasma concentrations of homocysteine would modify the established association between postoperative plasma concentration of CRP and postoperative delirium in patients.

Preoperative homocysteine and postoperative CRP were evaluated in this study because postoperative CRP is an established blood biomarker of postoperative delirium (Dillon et al., 2017; Vasunilashorn et al., 2017; Slor et al., 2019). However, the role of preoperative homocysteine in postoperative delirium has not been fully revealed (Kim et al., 2015; Weerink et al., 2018; Vahdat Shariatpanahi et al., 2019; Guo et al., 2020). Moreover, we chose homocysteine and CRP, but not other inflammatory factors, e.g., S100β and interleukin 6, in the present study because we aimed to use preoperative homocysteine and postoperative CRP to specifically investigate the effects of interaction between the predisposing factor (preoperative homocysteine) and the precipitating factor (postoperative CRP) on the postoperative delirium.

## Materials and methods

### Study design

We performed a prospective observational cohort study from June 22, 2016 to May 5, 2017, at Shanghai Tenth People’s Hospital, a university-affiliated hospital in Shanghai, P. R. China. The study protocol was approved by the hospital’s Human Research Ethics Committee (SHSY-IEC-3.0/15-78/01) on May 12, 2016.

All participants provided written informed consent for the study before initiating any study procedures. This study is being reported following the Strengthening the Reporting of Observational Studies in Epidemiology (STROBE) criteria.

### Study population

We screened patients scheduled for knee/hip replacement surgeries. Participants were included if they: (1) were 60 years old or older; (2) spoke Mandarin Chinese; (3) had general anesthesia, and (4) were able to provide informed consent. Patients were excluded if they had any of the following: (1) pre-existing delirium assessed according to the Confusion Assessment Method (CAM) algorithm (Inouye et al., 1990); (2) prior neurologic diseases (e.g., dementia, Parkinson’s disease, multiple sclerosis or stroke) according to the International Statistical Classification of Diseases and Related Health Problems 10th Revision (ICD-10-version, 2016); (3) a history of mental disorders (e.g., major depressive disorder and schizophrenia) according to the Diagnostic and Statistical Manual of Mental Disorders, 4th edition (DSM-IV) (American Psychiatric Association, 1997); (4) had abnormal cognitive function at the time of enrollment, evidenced by a Mini-Mental State Examination (MMSE) score above an education-adjusted threshold (18 for individuals with no school education, 20 for 1–6 years of education, or 24 for 7 or more years of education (Li et al., 2016), (5) pre-existing fever/infection, (6) significant past medical history, or (7) unwillingness to comply with the assessments.

### Preoperative interview

Preoperative assessments were performed one day before the scheduled surgery by well-trained researchers following a standard protocol. Participant characteristics were collected, including age, sex, education, body mass index (BMI), and Charlson Comorbidity Index (CCI) (Charlson et al., 1994). Preoperative cognitive function was assessed using the MMSE (Chinese version) (Li et al., 2016). The CAM algorithm (Inouye et al., 1990) was also performed preoperatively to exclude patients with pre-existing delirium. The Visual Analog Scale (VAS, ranging from 0 to 10) was used to assess subjective pain the day before surgery.

### Anesthesia and surgery

All participants underwent a hip or knee replacement under general anesthesia. The preoperative fasting, anesthetics used, airway management, opioid and neuromuscular blocking agent (and the reversal) usage, intravenous fluid administration, and mechanical ventilation were performed per the hospital policy and at the anesthesiologist’s discretion. The participants generally received 1–2 mg midazolam preoperatively. Anesthesia was induced with propofol (2 mg/kg), sufentanil (0.5–1 μg/kg), and cisatracurium (0.5 mg/kg) and was maintained with anesthetic sevoflurane or propofol. We obtained information regarding the American Society of Anesthesiologists (ASA) Physical Status Classification System (ASA Physical Status Classification System, 2014), surgery types, length of anesthesia duration, and length of surgery duration by reviewing the anesthesia records of the participants. All the patients received the standard postoperative analgesia. Moreover, the VAS was used to assess subjective pain on postoperative days 1, 2, and 3.

### Specimen collection and measurements

We collected 4 ml of venous blood during the insertion of intravenous catheters before the anesthesia and surgery in all participants to measure preoperative plasma concentrations of homocysteine. The blood sample was collected in anticoagulant tubes and was immediately centrifuged to collect the supernatant plasma. The plasma was stored in a −80°C freezer until measurement. Preoperative plasma concentration of homocysteine was determined by using the Roche Cobas 8000 system (Roche Diagnostic, Rotkreuz, Switzerland) with the enzyme cycling method.

Postoperative measurement of blood CRP was part of the clinical care of the patients. Thus, we obtained the postoperative blood CRP concentrations by checking the participants’ medical records. If participants had several postoperative CRP measurements, the concentration of the first postoperative measurement of plasm CRP was used for the final data analysis in the present study. Since the postoperative CRP measurement was part of the routine postoperative clinical care, the time of the postoperative blood collection (i.e., postoperative day) was not fixed on a particular day. Notably, in the clinical laboratory, the postoperative plasma concentrations of CRP were measured using an immunonephelometric method on a Nephelometer BNII (Siemens Healthcare, Germany), measuring a range from 3–200 mg/L.

### Assessment of delirium incidence

The primary outcome was the presence of delirium at any postoperative assessments performed in the first three days postoperatively. Postoperative delirium was determined by daily interviews on postoperative days 1, 2, and 3 using the CAM (Inouye et al., 1990). Each participant was assessed with CAM twice daily between 8–10 am and 4–6 pm. Patients were considered delirious if delirium was present on any of these assessments. Patients discharged prior to day three were excluded from the analysis. Participants were included in the data analysis as long as they were assessed for delirium at least once per day following surgery. We did not use CAM-ICU in the present study.

### Assessment of delirium severity

The severity of delirium was assessed as a secondary outcome. The severity of postoperative delirium was quantified using the CAM-Severity (CAM-S) long-form (Inouye et al., 2014), comprising 10 items capturing delirium features (range from 0 to 19). The Chinese version of CAM-S has demonstrated good reliability and validity among Chinese older adults (Mei et al., 2019). The peak (worst) CAM-S scores across all postoperative days were used to assess delirium severity for the data analysis (Vasunilashorn et al., 2020).

### Estimation of sample size

We estimated that 140 participants would provide 90% power to detect the potential difference in postoperative plasma concentration of CRP between the participants with and without postoperative delirium at a 5% significance level. This estimation was based on a previous study of elderly patients aged 65 years old. The patients who developed postoperative delirium had higher postoperative CRP concentrations than those who did not: 10.26 ± 5.81 mg/dL versus 6.96 ± 4.89 mg/dL (Lee et al., 2011).

According to our previous study, the estimated incidence of postoperative delirium was 25.6% (Shi et al., 2014), so we set the ratio of the participants who developed postoperative delirium to normal participants as 1 to 3. We set an estimated drop-out rate of 15% during postoperative assessment (Sakpal, 2010). Thus, we determined that we should enroll 165 participants in the study to have 140 participants for the final data analysis.

### Statistical analysis

The study included the data analysis of plasma concentrations of preoperative homocysteine, postoperative CRP, and the incidence and severity of postoperative delirium. Notably, all postoperative CRP measurements were performed as part of routine clinical care, mostly occurring within 1–3 days after surgery. The statistical analysis plan was finalized before analyzing the data.

Normally distributed continuous variables (e.g., BMI) and non-normally distributed continuous variables (e.g., the plasma concentration of homocysteine and CRP) are presented as the mean ± standard deviation (SD) and median [interquartile range (IQR), 25th–75th], respectively. As appropriate, the differences between patients who did and did not develop delirium were assessed with an independent samples *t*-test or Mann–Whitney *U* test. Categorical variables (e.g., sex, ASA, and CCI) are presented as frequencies and proportions and assessed with *chi-square* or *Fisher’s exact* test.

Preoperative homocysteine and postoperative CRP were scaled using z-scores in all models for analysis, which were calculated by subtracting the mean from an individual raw score and then dividing the difference by the standard deviation. The association between preoperative plasma concentration of homocysteine, the postoperative plasma concentration of CRP, and postoperative delirium incidence were assessed using logistic regression. Results are presented as odds ratio (OR) per one standard deviation change in the biomarker and their associated 95% confidence intervals (CI). The association between the biomarkers and the worst delirium severity was estimated using linear regression, with results reported as a mean difference [beta coefficient (β)] and it’s associated 95% CI. Model fit assuming a Gaussian distribution was evaluated by examining the residuals and model calibration.

The association between preoperative homocysteine and postoperative plasma concentration of CRP was assessed for both the primary (delirium incidence) and secondary (delirium severity) outcomes. Both predictors were evaluated in separate univariate models with each of the main effects, one model including postoperative CRP and preoperative homocysteine, and the final model including the main effects and the inclusion of an interaction term between preoperative homocysteine and postoperative CRP together. In order to visualize the interaction between postoperative CRP and preoperative homocysteine, the relationship between postoperative CRP and the predicted probability of postoperative delirium was reported for different preoperative plasma concentrations of homocysteine, namely the 10th, 25th, 50th, 75th, and 90th percentiles. Multivariable models were then created to adjust the associations between the biomarkers and outcomes for age, sex, preoperative MMSE, and day of postoperative CRP measurement for both the primary and secondary outcomes. These variables were selected for the adjustment based on previous studies as deemed clinically relevant while still considering model parsimony. Pearson Correlation was used to determine the relationship between pre-operative homocysteine and postoperative CRP. SPSS version 22.0 (SPSS Inc., Chicago, IL, United States) and R (version 4.0.5, Vienna, Austria) were used to analyze the data. Two-sided *p*-values less than 0.05 were considered statistically significant for all analyses.

## Results

### Participant characteristics

**FIGURE 1:**
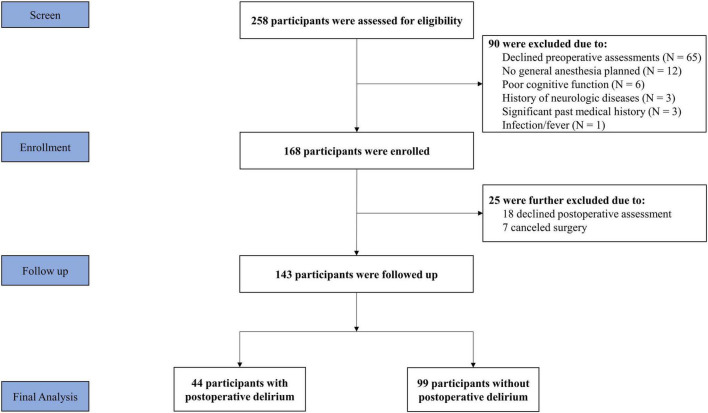
Flow Diagram. Two hundred and fifty-eight participants were initially screened for the study. Ninety participants were excluded, resulting in 168 participants included at the enrolment of the study. During the follow-up assessment, another 25 participants were lost. Thus, a total of 143 participants were included in the final data analysis.

A total of 258 patients were screened for inclusion in the study. Among them, 90 participants were excluded because they declined the required preoperative assessment of delirium (*N* = 65), had abnormal cognitive function (*N* = 6), did not plan to have general anesthesia or changed anesthesia (*N* = 12), had prior neurologic diseases (*N* = 3), had an infection/fever (*N* = 1), and had a significant past medical history (*N* = 3). Thus, 168 participants were enrolled in the study. After obtaining the consent, 25 participants were excluded because they were not interested in further participation in the study (*N* = 18), or canceled surgery (*N* = 7). Participants in the present study did not include patients who took psychoactive drugs to treat mental disorders or neurological diseases. No major complications occurred during the immediate postoperative period. There were no missing data for variables of interest in the current study. The final data analysis included 143 participants [69% female, median age 71 (IQR: 67–76) years] who underwent knee (88.0%) or hip (12.0%) surgery (Figure 1).

Notably, postoperative CRP was measured on different days. However, there was no statistically significant difference in the percentage of participants on each of the postoperative days when the postoperative plasma concentrations of CRP were measured between the cohort with postoperative delirium and the cohort without postoperative delirium (Supplementary Figure 1).

Delirium was assessed twice daily up to postoperative day three. Among the 143 included participants, 135 (94.4%) patients received all six assessments, seven (4.9%) participants received five assessments, and one (0.7%) participant received four assessments. Forty-four of the 143 participants (31%) developed postoperative delirium. There were no statistically significant differences in demographic, clinical characteristics, type of surgery, anesthesia, and other perioperative factors between participants with (*N* = 44) and without (*N* = 99) postoperative delirium, except for the length of surgery duration, the preoperative and postoperative day 2 VAS scores (Table 1).

**TABLE 1:** Clinical characteristics of the participants with and without postoperative delirium.

Variables	With postoperative delirium	Without postoperative delirium	*P*-value
	(N = 44)	(N = 99)	
**Demographic variables**			
Sex, Female, n (%)	29 (66)	70 (71)	0.566
Male, n (%)	15 (34)	29 (29)	
Age, median (IQR, 25th–75th), yr.	71 (68–79)	70 (66–75)	0.387
Education, median (IQR, 25th–75th), yr.	9 (5–12)	9 (6–12)	0.660
**CCI, n (%)**			
2	10 (23)	26 (26)	0.330
3	11 (25)	36 (36)	
4	15 (34)	21 (21)	
≥ 5	8 (18)	16 (16)	
BMI, mean ± S.D., kg/m^2^	26.15 ± 4.10	25.92 ± 3.37	0.741
Type of surgery, n (%) Keen surgery	36 (81)	90 (91)	0.161
Hip surgery	8 (19)	9 (9)	
**ASA, n (%)**			
I	1 (2)	5 (5)	0.194
II	31 (71)	79 (80)	
III	12 (27)	15 (15)	
Length of anesthesia, mean ± S.D., minutes	134 ± 40	131 ± 51	0.773
Length of surgery, mean ± S.D., minutes	102 ± 33	91 ± 30	0.042
Preoperative MMSE, median (IQR), scores	26 (23–27)	26 (24–28)	0.094
Peak CAM-S scores, median (IQR), scores	6 (5–7)	3 (2–4)	< 0.001
Preoperative VAS Scores, median (IQR)	8.0 (6.0–9.0)	6.0 (5.0–8.0)	0.027
Postoperative day 1 VAS Scores, median (IQR)	7.5 (6.0–8.0)	6.0 (4.0–8.0)	0.055
Postoperative day 2 VAS Scores, median (IQR)	6.0 (6.0–8.0)	6.0 (4.0–8.0)	0.017
Postoperative day 3 VAS Scores, median (IQR)	6.0 (5.0–8.0)	5.0 (4.0–7.0)	0.064
**Plasma variables**			
Preoperative homocysteine, (IQR, 25th–75th) (mmol/L)	15.43 (12.23, 21.26)	12.51 (10.42, 16.32)	0.036
Postoperative CRP, median (IQR, 25th–75th), (mg/L)	61.60 (40.48, 106.31)	46.50 (27.60, 78.20)	< 0.001

ASA, American Society of Anesthesiologists; BMI, Body-Mass-Index; CCI, Charlson comorbidity index; CAM-S, Confusion Assessment Method—Severity; CRP, C-Reactive Protein; MMSE, Mini-Mental State Examination; VAS, Visual Analog Scale. Normally distributed continuous variables were presented as Mean ± standard deviation (S.D.). An Independent t-test evaluated the difference; non-normally distributed continuous variables were presented in the median and interquartile range (25th to 75th percentile). The difference was assessed by Mann–Whitney test. The Chi-square test or Fisher’s exact test was used to determine the difference in the categorical variables.

Participants who developed postoperative delirium had higher median preoperative plasma concentration of homocysteine [15.43 (12.23, 21.26) mmol/L versus 12.51 (10.42, 16.32) mmol/L, *P* = 0.036] and postoperative plasma concentration of CRP [61.60 (40.48, 106.31) mg/L versus 46.50 (27.60, 78.20), mg/L, *P* < 0.001] than the participants without postoperative delirium (Table 1).

### Associations among homocysteine, C-reactive protein, and incidence of postoperative delirium

The postoperative plasma concentration of CRP was associated with the incidence of postoperative delirium before and after adjusting for age, sex, preoperative MMSE, and day of postoperative CRP measurement (adjusted OR per one standard deviation change in CRP: 1.51; 95% CI: 1.05–2.16; *P* = 0.026; Table 2). However, the preoperative plasma concentration of homocysteine was not associated with the incidence of postoperative delirium (Table 2).

**TABLE 2:** The association between the incidence of postoperative delirium and the preoperative homocysteine, postoperative C-reactive protein, or their interaction.

	OR (95% CI)	*P*-value	OR (95% CI)	*P*-value	OR (95% CI)	*P*-value
	*Univariate*		*Main effect*		*Interaction effect*	
**Postoperative delirium incidence before adjustment**						
Postoperative CRP	1.47 (1.04, 2.09)	0.030	1.43 (1.01, 2.04)	0.047	1.53 (1.06, 2.22)	0.023
Preoperative homocysteine	1.34 (0.94, 1.89)	0.102	1.28 (0.90, 1.83)	0.166	1.42 (0.98, 2.05)	0.064
Interaction of CRP and Homocysteine	N.A.		N.A		0.73 (0.53, 1.00)	0.051
**Postoperative delirium incidence after adjustment for age, preoperative MMSE, sex, and day of postoperative CRP measurement**						
Postoperative CRP	1.51 (1.05–2.16)	0.026	1.47 (1.02–2.12)	0.038	1.57 (1.07, 2.30)	0.020
Preoperative homocysteine	1.28 (0.88–1.87)	0.189	1.22 (0.83–1.79)	0.308	1.39 (0.93, 2.08)	0.126
Interaction of CRP and Homocysteine	N.A.		N.A.		0.71 (0.51, 0.99)	0.044

CI, confidence interval; CRP, C-reactive protein; N.A., Not applicable; OR, odds ratio; MMSE, Mini-Mental State Exam. The variables were scaled with Z scores in the models. Binary logistic regression was used to analyze the data, assuming an odds ratio of 1 under the null hypothesis.

**FIGURE 2:**
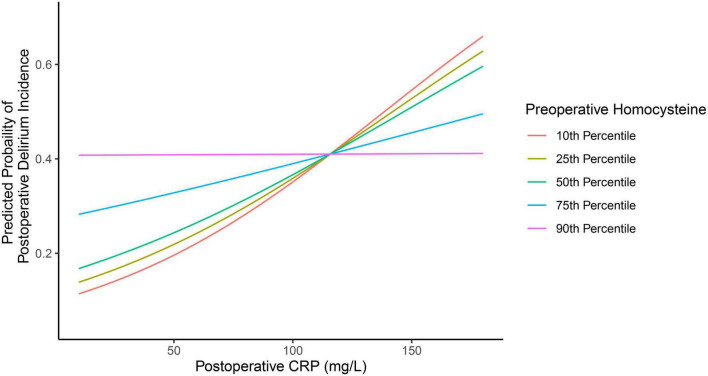
Different preoperative plasma concentrations of homocysteine lead to different associations between postoperative plasma concentrations of CRP and postoperative delirium incidence. There are different associations between the postoperative plasma concentration of CRP and the postoperative delirium incidence in the participants, with the preoperative plasma concentration of homocysteine at the 10th percentile, 25th percentile, 50th percentile, 75th percentile, and 90th percentile. The data suggest that the preoperative plasma concentration of homocysteine can modify the association between the postoperative plasma concentration of CRP and the postoperative delirium incidence. CRP, C-reactive protein.

Of note, a statistically significant interaction was observed between preoperative plasma concentration of homocysteine and postoperative plasma concentration of CRP on the incidence of postoperative delirium (*P* = 0.044, Table 2). Specifically, participants with lower preoperative homocysteine demonstrated a stronger association between postoperative CRP and postoperative delirium incidence than participants with higher levels of preoperative homocysteine. This is demonstrated in Figure 2, in which participants with 10th (9.23 mmol/L), 25th (10.81 mmol/L), or 50th percentile (13.16 mmol/L) of preoperative homocysteine plasma concentration demonstrated a stronger association between the postoperative plasma concentration of CRP and the postoperative delirium incidence when compared with participants in the 75th percentile (18.79 mmol/L). Further, in the participants with the 90th percentile (24.46 mmol/L) of preoperative homocysteine, the association between postoperative plasma concentration of CRP and postoperative delirium incidence disappeared. These data suggest that the association between the postoperative plasma concentration of CRP and the postoperative delirium incidence can be modified by the preoperative plasma concentrations of homocysteine.

Moreover, preoperative plasma homocysteine was not associated with postoperative plasma CRP before and after adjusting for age and sex (before adjustment: *R* = 0.125, *P* = 0.138; after adjustment: *R* = 0.126, *P* = 0.135; Pearson Correlation).

### Associations among homocysteine, C-reactive protein, and severity of postoperative delirium

We demonstrated that the participants with higher postoperative CRP concentrations were more likely to have higher CAM-S peak scores before and after adjusting age, sex, preoperative MMSE score, and day of postoperative CRP measurement (mean difference [β] in delirium severity per one standard deviation increase in CRP: 0.47 points; 95% CI: 0.18–0.76, *P* = 0.002, Table 3). However, there was no univariate, main, or interaction effect of preoperative plasma concentration of homocysteine on predicting the severity of postoperative delirium, suggesting that preoperative plasma concentration of homocysteine did not modify the association between postoperative plasma concentration of CRP and the severity of postoperative delirium.

**TABLE 3:** The association between the severity of postoperative delirium and the preoperative homocysteine, postoperative C-reactive protein, or their interaction.

	β Coefficient (95% CI)	*P*-value	β Coefficient (95% CI)	*P*-value	β Coefficient (95% CI)	*P*-value
	*Univariate*		*Main effect*		*Interaction effect*	
**Postoperative delirium severity before adjustment**						
Postoperative CRP	0.46 (0.15, 0.77)	0.004	0.45 (0.14, 0.76)	0.005	0.43 (0.12, 0.75)	0.007
Preoperative homocysteine	0.15 (−0.16, 0.47)	0.344	0.10 (−0.21, 0.40)	0.544	0.07 (−0.24, 0.39)	0.654
Interaction of CRP and Homocysteine	N.A.		N.A		0.10 (−0.17, 0.37)	0.466
**Postoperative delirium severity after adjustment for age, preoperative MMSE, sex, and day of postoperative CRP measurement**						
Postoperative CRP	0.47 (0.18, 0.76)	0.002	0.47 (0.17, 0.76)	0.002	0.45 (0.16, 0.75)	0.003
Preoperative homocysteine	0.10 (−0.22, 0.41)	0.546	0.03 (−0.28, 0.34)	0.832	0.00 (−0.32, 0.32)	0.981
Interaction of CRP and Homocysteine	N.A.		N.A.		0.09 (−0.17, 0.36)	0.476

β, beta; CI, confidence interval; CRP, C-Reactive Protein; MMSE, Mini-Mental State Exam; N.A., Not applicable. The variables were scaled with Z scores in the models. Linear regression was used to analyze the data, assuming a β Coefficient of 0 under the null hypothesis.

## Discussion

The data demonstrated an interaction of preoperative plasma homocysteine and postoperative plasma CRP on the incidence, but not severity, of postoperative delirium in patients. As a predisposing factor, the preoperative plasma homocysteine modified the established association between the postoperative plasma CRP, a precipitating factor, and the incidence of postoperative delirium. Specifically, the postoperative plasma concentration of CRP was associated with the postoperative delirium incidence in the patients with lower, but not higher, preoperative plasma concentrations of homocysteine. Pending further investigation, these data raise the possibility that preoperative blood homocysteine concentration may modify the relationship between postoperative blood CRP concentration and the incidence of postoperative delirium, suggesting that postoperative blood CRP amount may only predict postoperative delirium in specific, but not all, conditions.

We did not find an interaction between pre-operative plasma homocysteine and postoperative CRP on the severity of postoperative delirium. The reasons for such findings are not known at present. However, similar disassociation between incidence and severity of postoperative delirium has also been observed in previous studies (Larsen et al., 2010; Van Norden et al., 2021). Van Norden, J. et al. found that the peri-operative administration of dexmedetomidine was associated with a lower incidence, but not severity, of postoperative delirium (Van Norden et al., 2021). Larsen, K. A. et al. found that preoperative administration of olanzapine was associated with a significantly lower incidence of delirium but a longer duration and greater severity of postoperative delirium (Larsen et al., 2010).

A previous study showed that the participants with plasma concentrations of CRP greater than 235.73 mg/mL on postoperative day two were 1.5 times more likely to develop postoperative delirium with more severe symptoms (Vasunilashorn et al., 2017). Patients with postoperative delirium had a higher plasma concentration of CRP from postoperative days 2–5 (Slor et al., 2019). Consistently, we confirmed that the high postoperative plasma concentration of CRP was associated with postoperative delirium incidence and severity. Moreover, we further demonstrated that the established association between the postoperative plasma concentration of CRP and the postoperative delirium incidence could be modified by the preoperative plasma concentrations of homocysteine.

Preoperative plasma homocysteine is associated with postoperative delirium in patients after cancer surgery (Weerink et al., 2018) or hemiarthroplasty (Guo et al., 2020). However, another study showed no association between preoperative plasma homocysteine and the incidence of postoperative delirium in patients with cardiac surgery (Vahdat Shariatpanahi et al., 2019). Here, we showed that participants with postoperative delirium had higher median preoperative plasma concentrations of homocysteine than participants without postoperative delirium after hip or knee surgery. However, the preoperative plasma concentrations of homocysteine were not associated with the incidence or severity of postoperative delirium. Nevertheless, the present study showed that the preoperative plasma concentrations of homocysteine modified the established association between postoperative plasma concentration of CRP and postoperative delirium incidence.

The association between the postoperative plasma concentration of CRP and the incidence, duration, and severity of postoperative delirium can be modified by the apolipoprotein E (APOE) 4 genotype (Vasunilashorn et al., 2020) and the catechol-*O-*methyltransferase (COMT) genotype (Vasunilashorn et al., 2019). Specifically, the association between postoperative blood CRP concentration and postoperative delirium occurred in patients with APOE4, but not non-APOE4, carriers (Vasunilashorn et al., 2020), suggesting the contribution of gene-protein interaction to the development of postoperative delirium. In the present study, the association between the postoperative blood CRP concentration and postoperative delirium only occurred in patients with lower, but not higher, the preoperative blood concentration of homocysteine, suggesting the contribution of protein-protein interaction to the development of postoperative delirium.

Moreover, given that genotype is innate and cannot be modulated by intervention, our findings suggest a potential practical pathway for the intervention of postoperative delirium. We previously showed that preoperative dietary supplementation of VitB_12_ and folic acid could reduce cognitive impairment in aged mice by lowering homocysteine concentrations (Zhao et al., 2019). Thus, future studies could include exploring the possibility of reducing homocysteine concentrations as a potential targeted intervention for postoperative delirium.

Hyperhomocysteine causes BBB impairment (Tyagi et al., 2005; Kamath et al., 2006; Beard et al., 2011). CRP, produced by hepatocytes and then released into the blood (Sproston and Ashworth, 2018), can induce neuroinflammation and neurotoxicity (Song et al., 2015). Participants with hyperhomocysteine may have increased BBB permeability. Thus, mild inflammation, represented by a slight increase of postoperative plasma CRP, can promote postoperative delirium. On the other hand, participants without hyperhomocysteine may not have increased BBB permeability. Thus, greater postoperative concentrations of CRP are needed to develop postoperative delirium in the patients. More studies to test this hypothesis are warranted in the future.

There were several limitations of the present studies. First, the postoperative plasma concentrations of CRP were measured on different days postoperatively. However, there was no statistically significant difference in the percentage of participants whose postoperative plasma concentrations of CRP were measured on each of the postoperative days between the participants with postoperative delirium and the participants without postoperative delirium (Supplementary Figure 1). More importantly, the preoperative plasma concentration of homocysteine still modified the association between the postoperative plasma concentration of CRP and the incidence of postoperative delirium after adjusting the days when postoperative CRP amounts were measured (Table 2). Therefore, the conclusion that preoperative plasma concentration of homocysteine modified the association between the postoperative plasma concentrations of CRP and postoperative delirium incidence was not changed by the fact that postoperative plasma concentrations of CRP were measured on different days after the anesthesia/surgery. Second, 69% of the participants in the present study were female, and most had knee surgery. Thus, the present study’s findings need to be validated for broader applicability to male patients and other types of surgery. Third, it should be noted that the patients with postoperative delirium had worse pre- and postoperative pain scores than those without postoperative delirium. These data suggest pain can contribute to postoperative delirium, consistent with the results from the previous studies (Vaurio et al., 2006; Ma et al., 2022). Finally, the patients who developed postoperative delirium had a longer duration of surgery but not a longer duration of general anesthesia. These findings suggest that surgery contributes more to the development of postoperative delirium, consistent with the results from the recent studies that there was no significant difference in the incidence of postoperative delirium between the patients who underwent surgery with general anesthesia and the patients who underwent surgery with regional anesthesia (Neuman et al., 2021; Li et al., 2022).

In conclusion, the postoperative plasma concentration of CRP, but not the preoperative plasma concentration of homocysteine, was associated with the incidence and severity of postoperative delirium. However, the preoperative plasma concentration of homocysteine could modify the established association between the postoperative plasma concentration of CRP and the incidence of postoperative delirium. Pending further confirmative investigations, these findings suggest the regulation effects of predisposing factors on the established association of precipitating factors and postoperative delirium, promoting more research to reveal the pathogenesis, biomarkers, and targeted interventions of postoperative delirium in patients.

## Data availability statement

The data are available from the corresponding authors on reasonable request.

## Ethics statement

The studies involving human participants were reviewed and approved by the Human Research Ethics Committee of Shanghai Tenth People’s Hospital (SHSY-IEC-3.0/15-78/01) on May 12, 2016. The patients/participants provided their written informed consent to participate in this study.

## Author contributions

YS and ZX: study concept and design. XM, XCM, TT, MW, XW, HLZ, and JC: acquisition of data. HZ, KC, EM, ZX, and YS: analysis and interpretation of data. ZX, XM, and YS: drafting of the manuscript. KC, LX, and EM: critical manuscript revision for important intellectual content. XCM, LX, and YS: obtained funding. YS and XM: administrative, technical, and material support. YS: study supervision. All authors contributed to the article and approved the submitted version.
