# Supplementary material for: Preoperative homocysteine modifies the association between postoperative C-reactive protein and postoperative delirium
Source: Front Aging Neurosci. 2022 Sep 21;14:963421. doi: 10.3389/fnagi.2022.963421 (PMC9532549; doi:10.3389/fnagi.2022.963421)
Supplement: Supplementary file 1 [file Data_Sheet_1.docx]

Supplementary Material

**Preoperative homocysteine modifies the association between postoperative**

**C-reactive protein and postoperative delirium**

Xin Ma, Xinchun Mei, Tianyi Tang, Meijuan Wang, Xiaoyi Wei, Hailin Zheng, Jing Cao, Hui Zheng, Kathryn Cody, Lize Xiong, Edward R. Marcantonio, Yuan Shen, Zhongcong Xie

**Supplemental Figure 1. Measurement of postoperative plasma concentration of CRP at different days.**


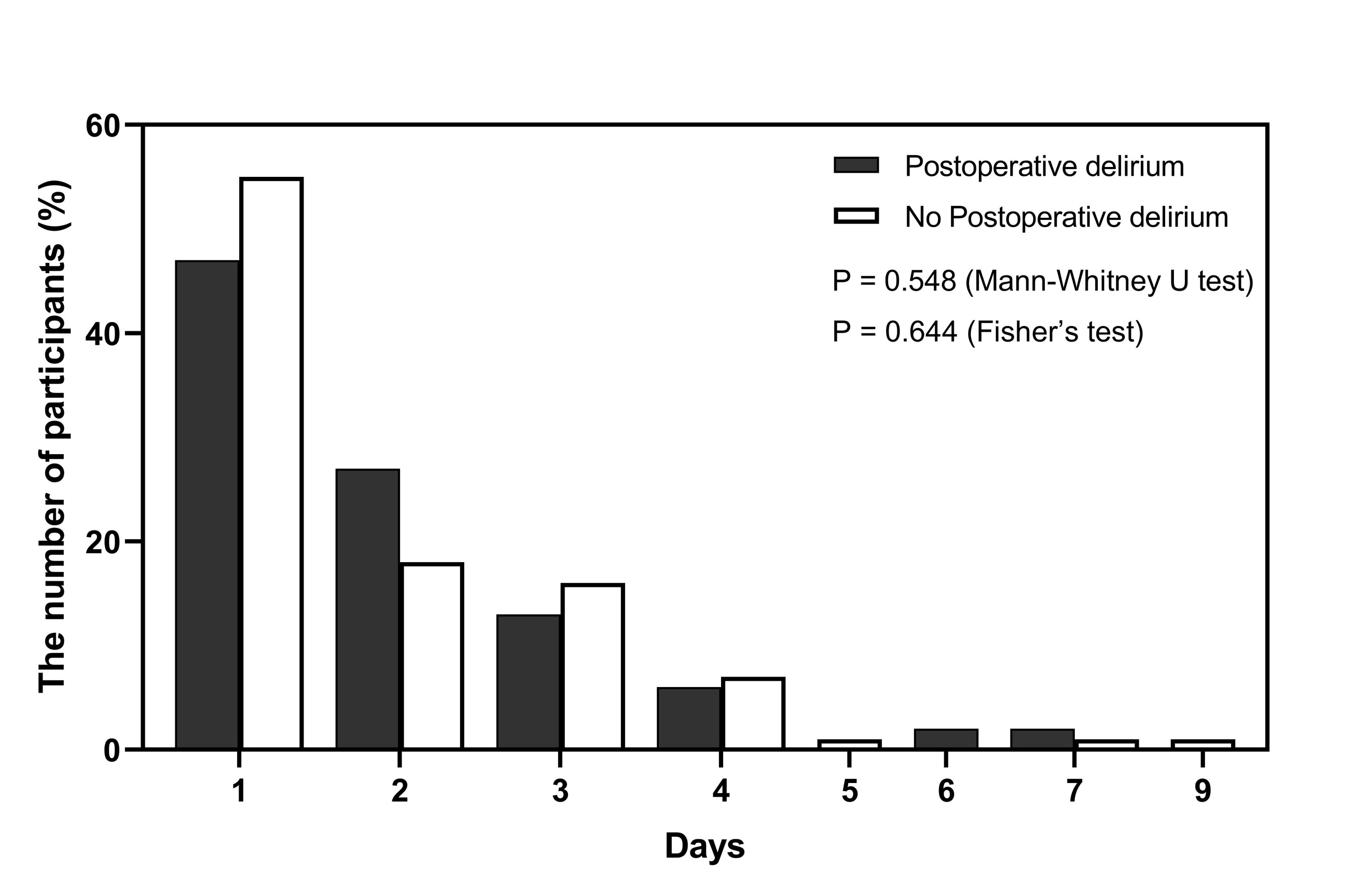


No difference was observed in the percentage of participants whose postoperative plasma concentrations of CRP were measured on each of the postoperative days between the participants with postoperative delirium and the participants without postoperative delirium. The bar represents the percentage of participants whose postoperative plasma concentrations of CRP was measured at different days after the anesthesia/surgery. Both Mann-Whitney U test and Fisher’s test were used to analyze the data. P values represent the difference in the percentage of participants whose postoperative plasma concentrations of CRP were measured on each of the postoperative days between the participants with postoperative delirium and the participants without postoperative delirium.

*Abbreviation: CRP, C-reactive protein.*
